# Validity and Reliability of a COVID-19 Stigma Scale Using Exploratory and Confirmatory Factor Analysis in a Sample of Egyptian Physicians: E16-COVID19-S

**DOI:** 10.3390/ijerph18105451

**Published:** 2021-05-19

**Authors:** Aya Mostafa, Nayera S. Mostafa, Nashwa Ismail

**Affiliations:** Department of Community, Environmental, and Occupational Medicine, Faculty of Medicine, Ain Shams University, Cairo 11566, Egypt; nayera_samy@med.asu.edu.eg (N.S.M.); nashwa_aly@med.asu.edu.eg (N.I.)

**Keywords:** COVID-19, stigma, scale, healthcare workers, factor analysis, reliability, validity, Egypt

## Abstract

Introduction: To date, a universal validated and specific tool for assessing coronavirus disease 2019 (COVID-19) stigma among healthcare workers is lacking. We adapted a SARS stigma scale that was developed using the Berger HIV scale for use as a COVID-19 stigma scale and evaluated its psychometric properties among Egyptian physicians. Methods: We administered the 17-item SARS stigma scale in an anonymous online questionnaire among 509 Egyptian physicians recruited via convenience sampling during a cross-sectional study in June 2020. Exploratory factor analysis was performed on half of the sample. Confirmatory factor analysis of the resulting model was done using structural equation modeling on the other half. Scale reliability was examined using Cronbach’s alpha for internal consistency. Convergent construct validity was assessed using regression models to examine the association between the adapted COVID-19 stigma scale and relevant factors. Results: Exploratory factor analysis yielded 16 items (E16-COVID19-S) that supported a three-factor structure: personalized stigma (8 items); concerns of disclosure and public attitudes (5 items); and negative experiences (3 items). Cronbach’s α was 0.909 for the total scale and 0.907, 0.663, and 0.789 for the three subscales. E16-COVID19-S was confirmed to have good model fit (comparative fit index = 0.964; root mean squared error of approximation = 0.056). E16-COVID19-S was independently associated with physicians’ younger age, lower qualification, working in an isolation hospital, and self-stigma, whether the scale was treated as categorical or continuous. Conclusions: E16-COVID19-S exhibited good internal consistency and construct validity among this sample of Egyptian physicians. These adequate psychometric properties make the E16-COVID19-S scale appropriate for use by researchers and practitioners.

## 1. Introduction

The coronavirus disease 2019 (COVID-19) pandemic has been associated with various forms of stigma and discrimination since its beginning, being first mislabeled as the ‘Chinese virus’ [1]. During the progressing pandemic, many incidents of physical and verbal attacks have been reported against specific populations such as healthcare workers (HCWs) in the United States of America, Italy, France, India, Bangladesh, Mexico, Malawi, as well as in Egypt [1,2,3]. Besides its psychological impact, COVID-19 stigma may play a role in spreading the infection or in poor prognosis because individuals carrying the disease may be concerned about disclosing their illness; hence, they may hide their symptoms or delay seeking medical care to avoid discrimination [4]. There are many factors that have led to what might be described as a parallel COVID-19 stigma pandemic, such as its continuously evolving nature and the emergence of new strains, incomplete understanding of its modes of transmission, public mistrust in conflicting measures and messages by authorities, uncertainty about the effectiveness of its preventive and treatment agents, the associated social restrictions, the prolonged duration of the pandemic, and its accompanied morbidity and mortality [1,5].

Being at the frontline of the pandemic response, HCWs have been particularly affected by the COVID-19 pandemic and its associated distress [6]. Approximately 10% of the 133.5 million globally reported COVID-19 cases to date are HCWs [7,8]. As a result, an emerging body of research on the psychological/mental health of HCWs has been compiling [9]. HCWs have faced many experiences or practices of COVID-19 stigma in the form of social avoidance, house eviction, restrictions from neighbors, public attacks, or insults [2]. For instance, ‘self-stigma’ was experienced when HCWs internally adopted the negative feelings or beliefs of others about their role in the COVID-19 pandemic and ‘anticipated stigma’ was the HCWs’ expectation of being stigmatized if their occupation became recognized [10]. Moreover, HCWs faced stigma practices, including discriminatory behavior such as social avoidance during daily activities or marginalization from community participation [10]. Research that quantitatively examined the extent of COVID-19 stigma among HCWs is limited. Studies from Nigeria [11], Pakistan [12], and India [13] reported that 28.3%, 41.9%, and 70% of studied HCWs experienced some form of stigma, respectively. However, these and other studies assessed COVID-19 stigma with non-specific/non-validated tools or among quarantined HCWs or non-HCWs [11,12,13,14,15,16,17,18].

As of 10 April 2021, Egypt had 208,082 COVID-19 cases and 12,323 deaths [8]; 427 deaths were physicians [19]. As in other global incidents, some Egyptian HCWs were spurned in fear of acquiring the infection [3]. In April 2020, anxious residents prevented the burial of a physician who died from COVID-19 in their village [20]. One year later, in April 2021, it was reported that the body of a nurse who died due to COVID-19 was exhumed and burnt in its grave by unknown men [21]. These stigmatizing practices against HCWs caused psychological distress to the families of these victims, stirred public criticism, and were condemned by higher local and religious authorities and by the media.

Mitigating COVID-19 social stigma is a timely necessity for upholding the psychological wellbeing and minimizing unfortunate outcomes among this much needed taskforce. In this context, early identification of COVID-19 stigma victims in this highly prone HCW population is crucial to enable prompt implementation of targeted interventions. To this end, a universal, validated COVID-19 stigma-specific tool is required for use by practitioners and researchers. However, such a tool is lacking locally and globally. To address this gap, we adapted a Severe Acute Respiratory Syndrome (SARS) stigma scale [22] that was originally developed using the Berger Human Immunodeficiency Virus (HIV) scale [23] and evaluated its psychometric properties in terms of validity and reliability among Egyptian physicians for use as a COVID-19 stigma scale.

## 2. Methods

Study design, procedures, and participants have been previously described in detail [3]. Briefly, 509 Egyptian physicians voluntarily participated in a cross-sectional study via convenience sampling during June 2020, at the peak of the first COVID-19 epidemic wave in Egypt. They accessed the link to the online self-administered questionnaire via social networks and emails and shared it with workmates. Responses were anonymous and participants were not provided with incentives. After providing informed consent, participants completed the three sections of the questionnaire, which have been detailed elsewhere [3]. In this article, we present the measures used to assess perceived COVID-19-related stigmatization and stigma experiences (including self-stigma and anticipated stigma).

### 2.1. Measures

#### 2.1.1. Perceived COVID-19-Related Stigmatization

At the time of the study, there were no specific published instruments that measured COVID-19 stigma. However, we found one tool that was developed by Verma et al. [22] that measured stigma in Chinese HCWs amid the 2004 SARS-related epidemic in Singapore, a similar epidemic due to a respiratory virus. Verma et al. adapted their tool from the Berger HIV stigma scale [23]. The SARS stigma scale developed by Verma et al. consisted of 17 items under four subscales, where each of the 17 items had more than one subscale assignment: subscale 1 addressed personalized stigma (11 items); subscale 2 addressed disclosure concerns (4 items); subscale 3 addressed negative self-image (3 items); and subscale 4 addressed concern with public attitudes (10 items) [22]. The items included in Verma et al.’s SARS stigma scale, their subscale assignments, and the corresponding item numbers from Berger et al.’s HIV stigma scale are presented in Table 1. Each item had four possible responses distributed as 1-4 Likert scale points: “strongly disagree”, “disagree”, “agree”, and “strongly agree”. Thus, the total SARS stigma scale score ranged from 17 to 68; higher scores indicated higher stigma levels. Verma et al. used factor analysis to identify the four sub-scales [22]. However, details of the scale structure were not available in the published article [22]. Therefore, we requested the SARS stigma scale and its scoring system from Verma and colleagues.

We adapted the SARS stigma scale to measure COVID-19 stigma among this sample of Egyptian physicians. Experts in public health, occupational medicine, and psychiatry assessed the face and content validity of the adapted COVID-19 stigma scale. The scale was administered in English because Egyptian physicians are familiar with the language and they study Medicine in English. The scale was piloted in 10 physicians to assess whether the questions and answer categories were clear and appropriate. Based on feedback from physicians in the pilot study, one modification was performed to rephrase the word “touching me” to “getting close to me”. This analysis did not include pilot data.

#### 2.1.2. COVID-19-Related Stigma Experiences

We hypothesized that other aspects of COVID-19 stigma that were asked about in the questionnaire and were experienced by this sample of Egyptian physicians would be associated with their stigma score based on our previous analysis and previous literature [3,12,13,14]. These measures were used to examine the convergent construct validity of the newly adapted COVID-19 stigma scale score, which included: age of physicians; physician’s qualifications; working in a COVID-19 isolation hospital; self-stigma (feelings of guilt for exposing physician’s family to COVID-19 infection); and anticipated stigma (the need to hide physician’s COVID-19 positive test result).

### 2.2. Ethical Considerations

This study was approved by the Ethical Review Board of Faculty of Medicine, Ain Shams University (FMASUR22/2020). All participants provided informed consent. Participation was voluntary, data were collected anonymously using serial study IDs, and data confidentiality was ensured.

### 2.3. Analysis

Exploratory factor analysis (EFA) was performed on one half of the sample and confirmatory factor analysis (CFA) on the other half to ascertain the factorial structure of the adapted COVID-19 stigma scale in this population of Egyptian HCWs. The sample was divided randomly using the automatic function in SPSS version 25. EFA was performed on the first dataset (n = 258) using principal component analysis. First, we checked the suitability of the data for factor analysis by performing the Kaiser–Meyer–Olkin (KMO) measure of sampling adequacy and Bartlett’s χ^2^ test of sphericity. The number of factors was inspected visually using the scree plot and was evaluated by examining the Kaiser Criterion of eigenvalues ≥1 [24] and the proportion of variance explained by each factor to identify the ideal number of latent factors [24]. Items were reserved in the factor structure if the strength of item loadings on factors was ≥0.4 [24]. Reliability was assessed by calculating Cronbach’s α to examine the internal consistency of the scale, factors, and items. Internal consistency values >0.7 are considered good scale reliability but may be reduced to >0.6 in exploratory studies [24]. Principal component analysis was repeated using Varimax rotation [24]. The item “I never feel the need to hide the fact that I am a HCW” was reverse scored according to the instructions provided by Verma et al. The resulting latent factors from the EFA were assessed conceptually.

Then, CFA was performed on the second dataset (n = 251) using structural equation modeling (IBM AMOS version 16) to examine the fit of the model resulting from the EFA. The maximum likelihood estimation method was applied. Covariance was included among each pair of factors in the model. Unstandardized and standardized estimates of regression weights as well as covariances and correlations between variables were obtained, and modification indices were examined. As the data were non-normally distributed, we performed Bollen–Stine bootstrapping to test the null hypothesis that the model is correct. We report indices of model fit [24]: the Tucker–Lewis index (TLI), the comparative fit index (CFI), the root mean square error approximation (RMSEA), and the p-value for a close fit (PCLOSE). Model fit was considered good if the CFI > 0.90, TLI > 0.90, RMSEA < 0.05 [24]. The ratio of chi-square on degrees of freedom (χ^2^/df < 2.00) was also assessed [25]. There were no missing observations in the variables included in the CFA.

We conducted bivariable and multivariable analyses to examine convergent construct validity between the new COVID-19 stigma scale that was obtained from EFA and CFA (dependent variable) and its associations with the measures that were hypothesized to be associated with COVID-19 stigma (independent variables: self-stigma and anticipated stigma). The dependent variable was converted into percentages, and was then further categorized into three categories using tertiles (mild, moderate, and severe stigma), as Charles et al. proposed, because universal cut-off points that could be used for calculating stigma scores are lacking [26]. In the bivariable analysis, the dependent variable was entered as a categorical variable; associations with the independent variables were examined using the chi-squared test for categorical variables and the analysis of variance test (ANOVA) for continuous variables. In the multivariable analysis, the dependent variable was entered as a continuous variable into a generalized linear model with all independent variables: age, qualification, working in a COVID-19 isolation hospital; self-stigma and anticipated stigma. Beta coefficients and 95% confidence intervals (CI) are reported. For all analyses, a *p*-value ≤ 0.05 was used as the threshold for statistical significance.

## 3. Results

General characteristics of the study sample of 509 Egyptian physicians have been previously reported [3]. Participants’ median age (range 24 to 70 years) was 42.0 (IQR 33.0–48.0) years; they were mostly females (69.4%), married (74.3%), and with a postgraduate degree (87%). Around one quarter (27.1%) of the studied HCWs reported direct involvement in caring for COVID-19 patients and 3.7% worked in a COVID-19 quarantine hospital. Not all participants had undergone tests to check their infection status (25.1%); 14.8% of them had a positive test result.

### 3.1. Exploratory Factor Analysis

EFA was performed on the first half of the dataset. First, principal component analysis was performed, including all 17 items of the SARS stigma scale, using a cut-off point of 0.4 for factor loadings. Data were suitable for EFA as KMO was greater than 0.5 (0.916) and Bartlett’s test of sphericity was significant (<0.001). By examining the scree plot, a three-factor solution was suggested. Three factors yielded eigenvalues >1.0. A varimax rotation type of factor analysis was found to be suitable as the factors were orthogonal. The EFA was repeated with varimax rotation. Two items of the scale cross-loaded on Factors 1 and 2; their highest factor loadings were on Factor 1: “Some people who know I am a HCW have grown more distant” and “Most people nowadays are uncomfortable around a HCW”. One item cross-loaded on Factors 1 and 3; its highest factor loading was on Factor 1: “I have stopped socializing with some people because of their reactions to my being a HCW”. Next, reliability tests were done for the items that loaded on each factor and for each of the three factors. The reliability of Factor 2 was initially low (α = 0.365); removing the item that loaded on this factor, “I never feel the need to hide the fact that I am a HCW”, improved the reliability of Factor 2 (α = 0.663). EFA and reliability tests were repeated on the remaining 16 items of the scale. Factor loadings of the items on each of the three factors are shown in Table 2.

The reliability of the total 16-item COVID-19 stigma scale was α = 0.916. Eight items loaded on Factor 1 (α = 0.907), five items loaded on Factor 2 (α = 0.663), and three items loaded on Factor 3 (α = 0.789). This model explained 60.5% of the variance in the sample. Conceptually, the eight items loading on Factor 1 were related to personalized stigma; therefore, Factor 1 was labeled “personalized stigma”. The five items loading on Factor 2 were related to concerns of disclosure and public attitudes; thus, Factor 2 was labeled “concerns of disclosure and public attitudes”. Finally, the three items loading on Factor 3 were related to experiences that the physicians viewed as negative; hence, Factor 3 was labeled “negative experiences” (Table 2).

### 3.2. Confirmatory Factor Analysis

CFA conducted within the second dataset for this 16-item model displayed the following indices: χ^2^ (94) = 167.167, χ^2^/df = 1.778, CFI =0.964, TLI = 0.953, RMSEA = 0.0056 and *p*-value = 0.235. Thus, this model was confirmed to have good model fit. All 16 items demonstrated distinctive and salient loadings ranging from 0.540 to 0.885 (Table 3). We deemed the resulting 16-item model the “E16-COVID19-S”; the E indicates that these data originated in Egypt and the S denotes stigma.

### 3.3. The COVID-19 Stigma Scale “E16-COVID19-S” and Associated Factors

The total score of the 16-item COVID-19-stigma scale “E16-COVID19-S” could range from 16 to 64. The median score of the E16-COVID19-S scale was 39.0 (IQR: 34.0-43.0). By categorizing the continuous E16-COVID19-S scale into low, moderate, and high stigma levels using tertiles, approximately one third (157/509, 30.8%) of physicians reported a severe level of stigma. The mean E16-COVID19-S scale score was significantly higher among physicians reporting a severe level of stigma than among those reporting a mild or moderate level of stigma (Table 4).

Physicians who experienced moderate or severe levels of COVID-19 stigma were significantly younger than those who reported mild levels of COVID-19 stigma. A higher proportion of physicians with lower qualifications reported more severe stigma levels than those with higher qualifications (51.2% vs. 26.8%, *p* < 0.001). Physicians working in COVID-19 isolation hospitals were more likely to report a severe level of COVID-19 stigma compared with those working in other hospitals (73.7% vs. 29.2%, *p* < 0.001). Physicians who experienced self-stigma (feelings of guilt for exposing physician’s family to COVID-19 infection) and anticipated stigma (the need to hide physician’s COVID-19 positive test result) reported significantly higher rates of severe COVID-19 stigma than those who did not report self-stigma (32.5% vs. 14.9%, *p* = 0.002; and 42.0% vs. 28.5%, *p* = 0.041) (Table 4).

In the multivariable regression analysis, participants’ E16-COVID19-S scale score was negatively associated with older age (β = −0.08, 95% CI: −0.15, −0.01, *p* = 0.021) and higher qualification (β = −3.39, 95% CI: −5.31, −1.47, *p* = 0.001), and was positively associated with HCWs’ work in isolation hospitals (β = 4.83, 95% CI: 1.43, 8.23, *p* = 0.005) and physicians’ self-stigma (β = 5.16, 95% CI: 2.93, 7.34, *p* < 0.001). Anticipated stigma was not independently associated with the E16-COVID19-S score (Table 5).

## 4. Discussion

To the best of the authors’ knowledge, this is the first study to examine the validity and reliability of a COVID-19 stigma-specific scale among HCWs using exploratory and confirmatory factor analysis. Our sample comprised 509 physicians working in different Egyptian healthcare facilities and had direct and indirect involvement in COVID-19 care pathways. EFA in this population suggested a 16-item scale (E16-COVID19-S) with a three-factor structure: personalized stigma; concerns of disclosure and public attitudes; and negative experiences. CFA confirmed that the E16-COVID19-S scale had good model fit. Using this scale, around a third of the studied physicians were found to experience severe COVID-19 stigma levels.

To date, limited quantitative research exists on the magnitude of COVID-19 stigma among HCWs. Few studies reported varying proportions of COVID-19 stigma (ranging from 28 to 70%) [11,12,13]. However, the studies published so far have utilized non-specific or non-validated stigma tools [11,12,13,14,15]. Studies that attempted to use a specific validated COVID-19 stigma scale have done so involving either non-HCWs [17,18] or a particular group of HCWs who were quarantined for lengthy periods [16]. The latter developed a 12-item COVID-19 stigma scale by directly selecting items from the Berger HIV stigma scale based on group discussions [16]. The scale developers then tested the pre-selected items using EFA only, without performing CFA, and reported that their study was limited by the small sample size (n = 61) [16]. The 12 items represented three dimensions: disclosure concerns and personalized stigma; concerns about public attitudes; and negative self-image [16]. It was not reported in which language the scale was administered among these Vietnamese HCWs [16]. However, the published items and their wording was different [16] from the original HIV stigma scale [23] and from the SARS stigma scale [22], which we used to develop the E16-COVID19-S scale.

By contrast, the E16-COVID19-S scale has preserved the original wording of the SARS stigma scale and consequently the HIV stigma scale upon which it was based. Additionally, the E16-COVID19-S scale was administered in the original English language, which makes its utilization possible for a wider base of practitioners and researchers worldwide. The E16-COVID19-S scale largely resembles the scale it was adapted from, the SARS stigma scale [22]. However, there were three main differences. First, only one item was removed from the 17-item SARS stigma scale [22] to develop the E16-COVID19-S scale, implying that the scale was consistent in measuring stigma related to different respiratory virus epidemics. The item was “I never feel the need to hide the fact that I am a HCW” and its exclusion improved the reliability of its subscale. This item was originally assigned under “disclosure concerns” in the SARS stigma scale; being negatively worded, it may have not been perfectly aligned with other items measuring the same subscale. Second, while the SARS stigma scale has four domains (personalized stigma; disclosure concerns; negative self-image; and concern with public attitudes), considering only the higher item subscale assignment reduces these four domains to only three, namely personalized stigma; disclosure concerns; and concern with public attitudes [22]. The E16-COVID19-S scale also consists of three domains but these were labeled slightly differently because three items fell under a unique domain, which we labeled ‘negative experiences’, although these were originally assigned to the ‘personalized stigma’ subscale in the SARS stigma scale [22]. The three items reflect certain negative reactions that HCWs experienced from others when the HCWs’ occupation became known to them. Therefore, labeling this domain as ‘negative experiences’ was deemed more suitable. Third, disclosure concerns and concerns with public attitudes were labeled collectively as one domain in the E16-COVID19-S scale instead of two separate domains as in the SARS stigma scale [22]. This seemed more in line with the EFA results regarding this domain’s items, which were similar in both scales, except for one item, “Being a HCW, I feel set apart and isolated from the rest of the world”, which was assigned under ‘concern of disclosure and public attitudes’ instead of ‘personalized stigma’ as in the SARS stigma scale [22]. Otherwise, the remaining items were assigned under similar domains in both scales.

The E16-COVID19-S scale demonstrated good convergent construct validity with factors that were hypothesized to be associated with COVID-19 stigma, whether the scale was represented in tertiles or as a continuous variable, suggesting that these results were robust. Younger physicians’ age, lower qualification, working in a COVID-19 isolation hospital, and self-stigma were also found to be associated with COVID19 stigma in previous studies [3,12,13,14]. However, age, education level, and work experience were not independently associated with experiencing incidents of COVID-19-related violence or harassment in other studies [12,15]. This may be due to the inclusion of other healthcare professions besides physicians in these studies. Notably, doctors were four-times more likely to experience all forms of COVID-19-related violence than other HCW occupations in Pakistan [12]. The authors attributed this higher liability to the greater involvement of doctors in the interaction with suspected and confirmed COVID-19 cases and in decision making [12].

## 5. Strengths and Limitations

This study presents a leading attempt to validate a specific COVID-19 stigma scale in HCWs. The sample consisted of physicians of various ages, work experience, and involvement in COVID-19 care pathways from different healthcare facilities in Egypt. The resultant E16-COVID19-S scale demonstrated acceptable psychometric properties: good model fit, high reliability, and convergent construct validity. The scale was administered in the English language, rendering it applicable for use in other settings, and was adapted from previous specific and validated stigma scales. These characteristics make the E16-COVID19-S appropriate for use in the early identification of stigmatized HCWs. Hence, it enables the implementation of prompt, targeted interventions to mitigate social stigma against HCWs.

However, the E16-COVID19-S scale needs further validation across different HCW populations to examine its robustness and whether there is a need to modify its items/constructs. More in-depth investigations, parallel qualitative assessments, random rather than non-random sampling methods, and larger samples including various HCW categories would help to confirm and generalize these findings. The cross-sectional study design did not enable us to investigate the long-term reliability (e.g., test–retest reliability) of this scale. Future studies in this field using a longitudinal research design with at least two measurement points could assess this aspect. This sample consisted of physicians only. It is yet to be studied whether this tool is suitable for use in other HCW occupations and whether it would therefore need translation to local languages to address them more adequately. Nonetheless, physicians were found to be more prone to COVID-19-related stigmatization than other HCW occupations in other studies [12]. Physicians also usually study medical sciences in English; thus, their familiarity with the English language may facilitate the E16-COVID19-S scale’s utilization. We adapted a scale designed to measure stigma related to another disease but both were respiratory infectious diseases that caused epidemics. Additionally, the limited research on COVID-19 stigma among HCWs prevents more detailed comparisons of our findings with the scale, degree, and determinants of COVID-19 stigma against HCWs in other settings. Future studies may address these limitations to broaden the scope of the scale’s application.

## 6. Conclusions

The E16-COVID19-S is the first validated, specific COVID-19 stigma scale among HCWs. Validation of a specific COVID-19 stigma scale is a demand in the current global pandemic. Amid the third wave of this COVID-19 pandemic, the parallel increase in morbidity and mortality among HCWs along with the soaring numbers of cases has led to higher exposure to stigma and discrimination. The E16-COVID19-S scale can help in identifying HCWs facing COVID-19 stigma; thus, it will assist in supporting HCWs among their communities and protecting their mental health and social wellbeing.

EFA in this population of Egyptian HCWs suggested a 16-item scale (E16-COVID19-S) with a three-factor structure: personalized stigma; concerns of disclosure and public attitudes; and negative experiences. CFA confirmed that the E16-COVID19-S scale had good model fit. The E16-COVID19-S scale was similar to the SARS stigma scale in its personalized stigma and disclosure concerns and concerns with public attitude domains. Almost one third of participating physicians experienced severe COVID-19 stigma. The E16-COVID19-S scale displayed good convergent construct validity with factors relevant to COVID-19 stigma such as younger physicians’ age, lower qualification, working in a COVID-19 isolation hospital, and self-stigma. The E16-COVID19-S scale demonstrated adequate reliability and validity and may assist practitioners and researchers in the early identification of HCWs who experience COVID-19 stigma to promptly facilitate their linkage to targeted interventions and services.

## Figures and Tables

**Table 1:** Items included in Verma et al.’s SARS stigma scale, their subscale assignments, and the corresponding item numbers from Berger et al.’s HIV stigma scale.

Similar Item No. (and Item Subscale Assignment)		The 17 Items Adapted by Verma et al. (2014) [22] for Measuring SARS-Related Stigma among HCWs
Berger et al. (2001) [23] HIV Stigma Scale	Verma et al. (2014) [22] SARS Stigma Scale	
32 (1,4)	14 (1,4)	People don’t want me around their children once they know I am a HCW
33 (1,4)	15 (1,4)	People have physically backed away from me when they learn I am a HCW
39 (1,4,3)	17 (1,3,4)	People seem afraid of me once they learn I am a HCW
28 (1,4)	12 (1,4)	Some people avoid getting close to me once they know I am a HCW
18 (1)	5 (1)	Some people who know I am a HCW have grown more distant
31 (1)	13 (1)	Some people close to me are afraid others will reject them if it becomes known that I am a HCW
35 (1)	16 (1)	I have stopped socializing with some people because of their reactions to my being a HCW
20 (4)	7 (4)	Most people nowadays are uncomfortable around a HCW
4 (2,4)	1 (2,4)	Telling someone I am a HCW is risky
9 (4)	2 (4)	HCW are treated like outcasts
17 (2)	4 (2)	I am very careful who I tell that I am a HCW
13 (1,3,4)	3 (1,3,4)	Being a HCW, I feel set apart and isolated from the rest of the world
19 (4,2)	6 (2,4)	I worry about people discriminating against me
26 (1)	10 (1)	I regret having told some people that I am a HCW
27 (1,4,3)	11 (1,3,4)	As a rule, telling others that I am a HCW has been a mistake
20 (4)	9 (1)	I have been hurt by how people reacted to learning I am a HCW
21 (2)	8 (2)	I never feel the need to hide the fact that I am a HCW

HCWs: Healthcare workers; 1: subscale 1 (personalized stigma); 2: subscale 2 (disclosure concerns); 3: subscale 3 (negative self-image); 4: subscale 4 (concern with public attitudes).

**Table 2:** Exploratory factor analysis of the adapted COVID-19 stigma scale among one half of the sample of Egyptian physicians (n = 258).

	COVID-19 Stigma Scale Items	Higher Subscale Assignment	Factor 1 *	Factor 2 *	Factor 3 *	Corrected Item-Total Correlation	Cronbach’s Alpha if Item Deleted
**1**	People don’t want me around their children once they know I am a HCW	1	0.825			0.686	0.900
**2**	People have physically backed away from me when they learn I am a HCW	1	0.804			0.732	0.898
**3**	People seem afraid of me once they learn I am a HCW	1	0.803			0.747	0.898
**4**	Some people avoid getting close to me once they know I am a HCW	1	0.787			0.730	0.898
**5**	Some people who know I am a HCW have grown more distant	1,2	0.662	0.414		0.636	0.902
**6**	Some people close to me are afraid others will reject them if it becomes known that I am a HCW	1	0.648			0.635	0.902
**7**	I have stopped socializing with some people because of their reactions to my being a HCW	1,3	0.645		0.414	0.643	0.901
**8**	Most people nowadays are uncomfortable around a HCW	1,2	0.544	0.470		0.608	0.903
**9**	Telling someone I am a HCW is risky	2		0.756		0.428	0.910
**10**	HCW are treated like outcasts	2		0.711		0.436	0.908
**11**	I am very careful who I tell that I am a HCW	2		0.615		0.545	0.905
**12**	Being a HCW, I feel set apart and isolated from the rest of the world	2		0.468		0.458	0.907
**13**	I worry about people discriminating against me	2		0.423		0.550	0.904
**14**	I regret having told some people that I am a HCW	3			0.840	0.595	0.903
**15**	As a rule, telling others that I am a HCW has been a mistake	3			0.812	0.540	0.905
**16**	I have been hurt by how people reacted to learning I am a HCW	3			0.673	0.520	0.905
	**Total % of variance explained/Eigenvalues**	60.466	6.986	1.396	1.292		
	**Cronbach’s alpha of total scale/subscales**	0.909	0.907	0.663	0.789		
	**KMO/Bartlett’s Test of Sphericity**	0.918	<0.001				

* Factor 1: Personalized stigma; Factor 2: Concerns of disclosure and public attitudes; Factor 3: Negative experiences.

**Table 3:** Confirmatory factor analysis showing standardized regression weights of the 16 items constituting the adapted COVID-19 stigma scale “E16-COVID19-S” among the second half of the sample of Egyptian physicians (n = 251).

COVID-19 Stigma Scale Items “E16-COVID19-S”		Factor 1 *	Factor 2 *	Factor 3 *
		Standardized Regression Weights		
1	People don’t want me around their children once they know I am a HCW	0.817		
2	People have physically backed away from me when they learn I am a HCW	0.817		
3	People seem afraid of me once they learn I am a HCW	0.848		
4	Some people avoid getting close to me once they know I am a HCW	0.781		
5	Some people who know I am a HCW have grown more distant	0.639		
6	Some people close to me are afraid others will reject them if it becomes known that I am a HCW	0.734		
7	I have stopped socializing with some people because of their reactions to my being a HCW	0.723		
8	Most people nowadays are uncomfortable around a HCW	0.540		
9	Telling someone I am a HCW is risky		0.584	
10	HCW are treated like outcasts		0.627	
11	I am very careful who I tell that I am a HCW		0.657	
12	Being a HCW, I feel set apart and isolated from the rest of the world		0.587	
13	I worry about people discriminating against me		0.651	
14	I regret having told some people that I am a HCW			0.885
15	As a rule, telling others that I am a HCW has been a mistake			0.786
16	I have been hurt by how people reacted to learning I am a HCW			0.686

* Factor 1: Personalized stigma; Factor 2: Concerns of disclosure and public attitudes; Factor 3: Negative experiences.

**Table 4:** The E16-COVID19-S scale score, levels, and associated factors for assessment of construct validity (N = 509).

	Total	COVID-19 Stigma Level					
		Mild	Moderate	Severe			
	N = 509	n = 23	n = 329	n = 157	Statistic	*V* ^a^	*p*-Value ^b^
**E16-COVID19-S scale, mean score ± SD**	38.5 ± 7.8	18.3 ± 1.9	36.0 ± 4.6	46.0 ± 3.7	F = 600.261		<0.001
**E16-COVID19-S scale, median score (IQR)**	39.0 (34.0–43.0)	18.0 (16.0–20.0)	37.0 (33.0–40.0)	46.0 (44.0–48.0)			
**Age (years)**	41.5 ± 10.2	49.0 ± 9.7	41.7 ± 9.8	40.1 ± 10.6	F = 8.042		<0.001
**Qualification, n (%)**	**n (column%)**	**n (row%)**	**n (row%)**	**n (row%)**			
Lower (MBBCh/diploma)	84 (16.5)	1 (1.2)	40 (47.6)	43 (51.2)	χ^2^ = 18.989	0.201	<0.001
Higher (Master/Doctorate)	425 (83.5)	22 (5.2)	289 (68.0)	114 (26.8)			
**Workplace, n (%)**							
Other	490 (96.3)	22 (4.5)	325 (66.3)	143 (29.2)	χ^2^ = 16.449	0.186	<0.001
Isolation hospital	19 (3.7)	1 (5.3)	4 (21.1)	14 (73.7)			
**Self-stigma, n (%)**							
No	47 (9.2)	6 (12.8)	34 (72.3)	7 (14.9)	χ^2^ = 11.440	0.157	0.002
Yes	462 (90.8)	17 (3.7)	295 (63.9)	150 (32.5)			
**Anticipated stigma, n (%)**							
No	421 (82.7)	19 (4.5)	282 (67.0)	120 (28.5)	χ^2^ = 6.288	0.112	0.041
Yes	88 (17.3)	4 (4.5)	47 (53.4)	37 (42.0)			

(^a^) *p*-values were computed using chi-squared test and Fischer’s exact test for categorical independent variables and analysis of variance for continuous independent variables. (^b^) Cramer’s V was used as a measure of effect size for the analyses with categorical variables.

**Table 5:** Regression analyses of the factors associated with E16-COVID19-S scale.

	Bivariable Association with E16-COVID19-S Scale				Multivariable Association with E16-COVID19-S Scale			
	β	95% CI		*p*-Value	β	95% CI		*p*-Value
		Lower	Upper			Lower	Upper	
**Age in years (increasing)**	−0.154	−0.220	−0.089	<0.001	−0.083	−0.153	−0.012	0.021
**Qualification**								
Lower (MBBch/diploma)	Ref				Ref			
Higher (Master/Doctorate)	−4.518	−6.306	−2.731	<0.001	−3.387	−5.305	−1.469	0.001
**Workplace**								
Other	Ref				Ref			
Isolation hospital	5.068	1.511	8.626	0.005	4.827	1.427	8.226	0.005
**Self-stigma**								
No	Ref				Ref			
Yes	5.251	2.948	7.553	<0.001	5.160	2.932	7.388	<0.001
**Anticipated stigma**								
No	Ref				Ref			
Yes	1.950	0.161	3.739	0.033	1.625	-0.077	3.328	0.061

## Data Availability

All relevant data are within the manuscript.
